# An Electrochemical Immunosensor for Sensitive Detection of Exosomes Based on Au/MXenes and AuPtPdCu

**DOI:** 10.3390/mi16030280

**Published:** 2025-02-27

**Authors:** Jie Gao, Rong Yang, Xiaorui Zhu, Jiling Shi, Sufei Wang, Aihua Jing

**Affiliations:** 1 School of Secondary Vocational Education, The Open University of China, Beijing 100031, China; gaoj@ouchn.edu.cn; 2 School of Medical Technology and Engineering, Henan University of Science and Technology, Luoyang 471023, China; 18113518547@163.com (R.Y.); 15903076659@163.com (J.S.); wangsufei2023@163.com (S.W.); 3 Collaboration Innovative Center of Henan Province for Energy-Saving Building Materials, Xinyang Normal University, Xinyang 464000, China; xrzhu@xynu.edu.cn

**Keywords:** AuPtPdCu, MXenes, early diagnosis, exosome detection, electrochemical biosensor, multi-metallic nanoparticles

## Abstract

Exosomes are important biomarkers for liquid biopsy in early cancer screening which play important roles in many biological processes, including apoptosis, inflammatory response, and tumor metastasis. In this study, an electrochemical aptamer immunosensor based on Au/MXene and AuPtPdCu was constructed for the sensitive detection of colorectal cancer-derived exosomes. AuNPs were deposited in situ on the surface of MXenes as a sensing platform due to their large specific area, excellent conductivity, and higher number of active sites for aptamer immobilization. The aptamer CD63 immobilized on Au/MXene can specifically capture target exosomes. Therefore, the AuPtPdCu-Apt nanoprobe further enhanced the sensitivity and accuracy of the immunosensor. A low limit of detection of 19 particles μL^−1^ was achieved in the linear range of 50 to 5 × 10^4^ particles μL^−1^ under optimal conditions. The immunosensor developed herein showed satisfactory electrochemical stability and anti-interference ability for the detection of exosomes in real serum samples.

## 1. Introduction

Cancer, heart disease, and diabetes are three major threats to human health, and cancer is typically regarded as an incurable illness [1]. Therefore, the early detection, diagnosis, and treatment of cancer are indispensable for saving the lives of cancer patients [2]. Tumor-derived exosomes retain specific information about a large number of biomolecules, (e.g., proteins and nucleic acids) from their parent tissues or cells, and are significant markers for the early diagnosis of cancer [3,4]. Exosome detection can be accomplished by testing specific exosome proteins, such as CD82 [5] and TRIM3 [6], or through the detection of circulating nucleic acids [7] such as miR-1910-3p and miR-200c [8]. Nucleic acid aptamers are nucleic acid sequences that can specifically bind to active small molecules [9]. Sensors for detecting exosomes based on aptamers, such as fluorescent aptamer sensors [10], electrochemical aptamer sensors [11], colorimetric aptamer sensors [12], and photoluminescence sensors [13], have been reported for the quantitative detection of exosomes with natural structures and functions. Furthermore, multiplexed near-field optical trapping techniques have been developed to tackle the heterogeneity of exosomes, providing a new approach for studying the heterogeneity of biological systems [14]. Among them, electrochemical aptamer sensors possess a wide detection range, low detection limit, and high sensitivity, due to improvements in nanotechnology and the utilization of advanced nanomaterials [15]. Hence, electrochemical aptamer sensors have significant application prospects in the clinical detection of early-stage cancers. Moreover, state-of-the-art exosome sensing devices fabricated with 3D plasmonic photonic crystals [16], box-like resonance shape dielectric gratings [17], and microfluidic photonic crystals [18] can enhance the performance of the proposed detection methods.

The sensing amplification of electrochemical biosensors for exosome identification and detection depends on the exosome recognition platform. Thus, such a platform should have a good capacity to immobilize more exosomes and enhance electron transfer. Therefore, materials with good electron transfer ability, biocompatibility, and a large specific surface area are considered suitable for immobilization [19]. Rocco’s research group has effectively utilized biochar with a porous structure and extensive surface area for the development of an electrochemical sensor, making outstanding contributions to the development of a carbon bioeconomy [20]. Novel MXenes (e.g., Ti_3_C_2_T_X_) have attracted the attention of researchers as supporting materials in electrochemical biosensors, due to their large specific surface area, high electrical conductivity [21], thermal conductivity [22], and good biocompatibility [23]. Due to their unique properties and advantages, MXenes have emerged as optimal materials in the sensor field for the detection of cancer biomarkers, such as carcinoembryonic antigens [24], extracellular vesicle exosomes [25], and MUCl [26]. Unlike most other two-dimensional materials, such as graphene [27], MXenes have high initial metal conductivity [28]. As a result, MXene-based strain sensors exhibit greater resistance during stretching [29]. Flexible pressure sensors prepared with MXenes are an integral part of wearable electronic devices and are widely used in health monitoring [30], human–machine interfaces [31], and robotics [32]. The surface of Ti_3_C_2_ has many active sites that can form stable chemical bonds with metal nanoparticles, enabling the construction of metal/MXene nanoparticle hybrids. Nanoparticle-functionalized MXene nanocomposites, such as Ag/MXene [33] and MXene/magnetic iron oxide nanocomposites [34], have demonstrated electrocatalytic activity suitable for biosensing applications. Au nanoparticles have been demonstrated to be excellent substrate materials for capturing antibodies and biomolecules in the fabrication of immunosensors [35]. Au nanoparticles are easily aggregated due to their small particle size and large electrochemically active areas. Therefore, it is better to decorate gold nanoparticles on supports such as 3D structures with high surface area, in order to maintain their activity. In this study, gold nanoparticles were decorated on 3D Ti_3_C_2_ to form Au/Ti_3_C_2_ with remarkable conductivity and large surface area, following which the Au/Ti_3_C_2_ was modified on an electrode as a substrate material for the capture of the CD63 aptamer for the purpose of binding the exosome protein CD63 for quantitative detection of exosomes.

Probe immobilization and signal carrier selection are key parts of sensor construction and are closely related to the analysis performance of a sensor [36], which should exhibit superior catalytic properties or act as an electroactive substance for sensing applications [37]. Multi-metallic nanomaterials have broad applications in probe fixation and as signal carriers due to their superior catalytic activity, excellent durability, good electrical conductivity, and large surface area [38]. For instance, PdPtRu trimetallic nanozymes have been fabricated as electrochemical immunosensors for sensitive SARS-CoV-2 antigen detection [39]. Dendritic AuPd@Au and AuPd networks have been used to construct immunosensors for PSA and CA153 detection [40]. AlCuFe quasicrystals and resin composite enable optical materials to operate efficiently across a wide spectral range while withstanding mechanical deformation [41]. The superior analytical performance of these sensors can primarily be attributed to the synergistic effects of and electronic interactions between multi-metals and the specialized nanostructures [42]. Therefore, new multi-metallic nanoparticles with particular structures are expected to provide a platform for the development of immunosensors with improved analytical signals.

**Figure 1:**
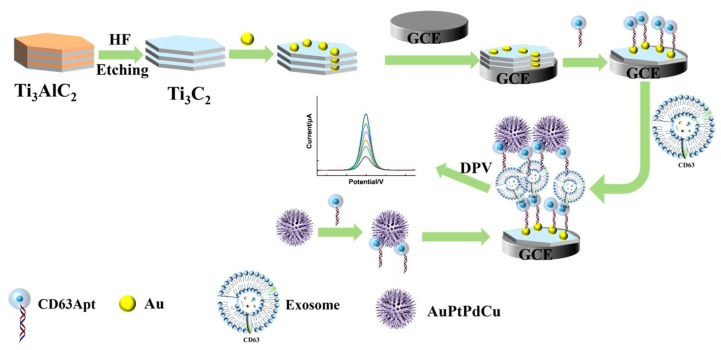
Preparation process of proposed sensor.

In this study, a sandwich-type electrochemical aptamer sensor based on Ti_3_C_2_ decorated with Au nanoparticles for the sensitive detection of exosomes was successfully fabricated. The fabrication process of the sensor is illustrated in Figure 1. Ti_3_C_2_ was chosen as the platform, and Au nanoparticles were then grown on MXene surfaces in situ to immobilize the CD63 aptamer, thus enhancing the performance of the biosensor. The CD63 aptamer immobilized on Au/Ti_3_C_2_ can specifically recognize exosomes and generate current signals for quantitative detection. Three-dimensional urchin-like AuPtPdCu nanomaterials were prepared for use as electrochemical nanoprobes. The unique urchin-like structure and four metal components of AuPtPdCu allowed for excellent catalytic performance and greatly enhanced the sensitivity of the electrochemical immunosensor. The fabricated biosensor exhibited a linear relationship within the exosome concentration range of 5 × 10^1^ particles μL^−1^ to 5 × 10^4^ particles μL^−1^, with a low detection limit of 19 particles μL^−1^ (S/N = 3). The developed electrochemical biosensor has clinical value for the early diagnosis of colorectal cancer.

## 2. Materials and Methods

### 2.1. Reagents and Instruments

Titanium aluminide (Ti_3_AlC_2_) was bought from Tianjin Feng chuan Reagent Company (Tianjin, China). Lithium fluoride, gold (III) chloride tetrahydrate, chloroplatinic acid hex hydrate, palladium chloride, ascorbic acid, and cetyltrimethylammonium bromide were obtained from Sangon Biotech (Shanghai, China). Silver nitrate was obtained from ThermoFisher Company (Shanghai, China). Sodium borohydride and copric chloride dihydrate were purchased from Macklin Biotech (Shanghai, China). Twice-distilled water was used to prepare phosphate buffers (PBS, 10 mmoL L^−1^, pH 7.4). Human serum was obtained from Luoyang Blood Center (Luoyang, China).

Scanning electron microscope (SEM, JEOL JSM-7800F, JEOL Ltd., Tokyo, Japan), transmission electron microscope (TEM, JEOL JTM-2100, JEOL Ltd., Tokyo, Japan), and X-ray diffraction (XRD, D8 ADVANCE X-ray diffractometer, Bruker AXS Ltd., Karlsruhe, Germany) techniques were used to characterize the products. A CHI660E workstation (Chenhua, Shanghai, China) with a three-electrode system was used to obtain the electrochemical detection data.

### 2.2. Preparation of Ti_3_C_2_

The multilayered Ti_3_C_2_ was synthesized according to a previously reported procedure with modification [43,44]. In brief, 2.0 g of Ti_3_AlC_2_ powder and 20 mL of HF (40 wt%) solution was gradually mixed at 0 °C for 10 min. This mixture was then stirred at 26 °C for 18 h until the reaction was completed. The mixture was washed until the pH was lower than 6. The obtained Ti_3_C_2_ was ultrasound-dispersed in water for further use.

### 2.3. Synthesis of Au/Ti_3_C_2_

Firstly, 0.6 mL of 1% HAuCl_4_ solution and 0.2 mL 0.2 moL L^−1^ K_2_CO_3_ were added to 50 mL of 0.4 mg mL^−1^ Ti_3_C_2_ suspension at 0 °C with stirring. Then, 0.4 mL of 0.5 mg mL^−1^ NaBH_4_ was added three times into the mixture. The mixture was stirred at 30 °C for 20 h. The Au/Ti_3_C_2_ was collected by performing centrifugation, washed with DMF and ethanol, and then dried in vacuum at 60 °C for 12 h [45].

### 2.4. Synthesis of AuPtPdCu

Briefly, 2.5 mL of 20 mmol L^−1^ PdCl_2_, 2.5 mL of 10 mmol L^−1^ H_2_PtCl_2_, 0.8 mL of 42 mmol L^−1^ HAuCl_4_, 1.0 mL of 20 mmol L^−1^ CuCl_2_, 200 mg of KBr, and 0.2 mL of 6 mmol L^−1^ HCl were mixed with 10 mL of 1% PEO solution to form a homogeneous suspension. Then 2.0 mL of 0.1 mol L^−1^ ascorbic acid (AA) solution was added to the above solution and reacted at 95 °C for 30 min. The resulting AuPtPdCu was centrifuged at 7000 rpm and washed several times with twice-distilled water and dried in a vacuum at 50 °C for further use [46].

### 2.5. Preparation of AuPtPdCu-Apt Nanoprobe

At 37 °C, 300 μL EDC (400 mM), NHS (100 mM), and 200 μL aptamer (-NH_2_) mixture were activated for 1 h, following which 500 μL of 1.0 mg mL^−1^ AuPtPdCu was added to the activated solution. AuPtPdCu-Apt was obtained after continuous incubation at 37 °C for 2 h, followed by centrifuge washing (12,000 rpm; 10 min) and dispersion with deionized water for AuPtPdCu-Apt [19].

### 2.6. Exosome Extraction

The HCT116 human colon cancer cell line (HCT-1165FR) obtained from the American Type Culture Collection was cultured to secrete exosomes [47]. The culture medium was centrifuged at 500× *g* for 15 min, at 2000× *g* for 20 min, and then filtered. The precipitates were then centrifuged twice (3000× *g*, 15 min) and then at 100,000× *g* for 90 min. Exosomes were dispersed in PBS solution and stored at −20 °C until further use.

The size of the exosomes (approximately 120 nm) was measured using a Malvern particle size analyzer (Zetasizer Nano ZS90, Shanghai, China). The concentration of exosomes (2.2 × 10^9^ exosomes µL^−1^) was determined using the BCA method.

### 2.7. Calculation of Analytical Parameters

The heterogeneous electron transfer constant (k^0^) for the reversible electrode probe [Fe(CN)_6_]^3−/4−^ was calculated with Equation (1) (Randles’ theory), Equation (2), and Equation (3) (Marcus’ theory) [48].

(1) $$k^{o}=\varphi \sqrt{\frac{D_{0}\pi \nu F}{RT}{\left(\frac{D_{Red}}{D_{Ox}}\right)}^{\alpha }}$$

(2) $$\varphi =\frac{\left(-0.6288+0.0021∆E\right)}{\left(1-0.0170∆E\right)}$$

where *D*_0_ is the average diffusion coefficient; *D_Ox_* and *D_Red_* are the diffusion coefficients for the ferricyanide/ferrocyanide redox reaction, respectively; *ν* is the scan rate (V/s); *n* is the number of electrons; *F* is the Faraday constant (mol^−1^); *T* is the temperature (K); *R* is the universal gas constant (J/Kmol); and *α* is the dimensional transfer coefficient.

The electron transfer constant (k^0′^) was calculated by Equation (3):

(3) $$k^{o\prime }=\frac{RT}{n^{2}F^{2}ACR_{ct}}$$

where A is the electrode surface (cm^2^), C is the concentration of the redox couple ferro–ferricyanide (mol/L), and *R_ct_* is the charge transfer resistance (Ω).

The limit of detection (LOD) is defined as the maximum signal attenuation corresponding to three times the standard deviation in the absence of exosomes.

The percentage recovery (*R*%) was calculated according to Equation (4):

(4) $$R\%=(\frac{x_{i}-x_{0}}{x_{s}})\times 100$$

where *x_i_* and *x*_0_ are the observed value and the actual value, and *x_s_* is the immunosensor response after the incubation of each analyte in the standard solution.

### 2.8. Construction of the Electrochemical Immunosensor

Figure 1 shows a schematic of the electrochemical biosensor fabrication process. First, a glassy carbon electrode (GCE, 3 mm in diameter) was carefully polished and washed to obtain a mirror-like surface in a standard manner. Subsequently, 10 μL of Au/Ti_3_C_2_ (1.0 mg mL^−1^) was dropped on the GCE and dried at 4 °C to obtain Au/Ti_3_C_2_/GCE. The electrode was then immersed in EDC/NHS (100 μM/300 μM) at 37 °C for 1 h. Next, 100 μM CD63 aptamer solution was mixed with 10 mM tris(2-carboxyethyl) phosphine (TCEP, Sigma-Aldrich, St. Louis, MO, USA) for 1 h, and CD63 aptamers were diluted to 1 μM with 4-(2-hydroxyethyl)-1-piperazineethanesulfonic acid (HEPES, Sigma-Aldrich, St. Louis, MO, USA) buffer. Thereafter, 10 μL of the prepared solution of CD63 aptamers was dipped on the surface of the Au/Ti_3_C_2_/GCE at 37 °C for 2 h to obtain Apt/Au/Ti_3_C_2_/GCE. The Apt/Au/Ti_3_C_2_/GCE was then washed twice with distilled water. Next, 30 μL of 1 mM MCH was added to the Apt/Au/Ti_3_C_2_/GCE at 37 °C for 2 h, in order to block the non-specific adsorption sites. To remove excess MCH, the Apt/Au/Ti_3_C_2_/GCE was washed thoroughly with PBS. The Apt/Au/Ti_3_C_2_/GCE was incubated with different concentrations of exosomes. Then, the exosomes/Au/Ti_3_C_2_/GCE were incubated with AuPtPdCu-Apt (10 µL) for 2 h at 37 °C to form AuPtPdCu-Apt/exosomes/Au/Ti_3_C_2_/GCE. Finally, the AuPtPdCu-Apt/exosomes/Au/Ti_3_C_2_/GCE was prepared for testing.

## 3. Results and Discussion

### 3.1. Characterization of Ti_3_C_2_ and Au/Ti_3_C_2_

**Figure 2:**
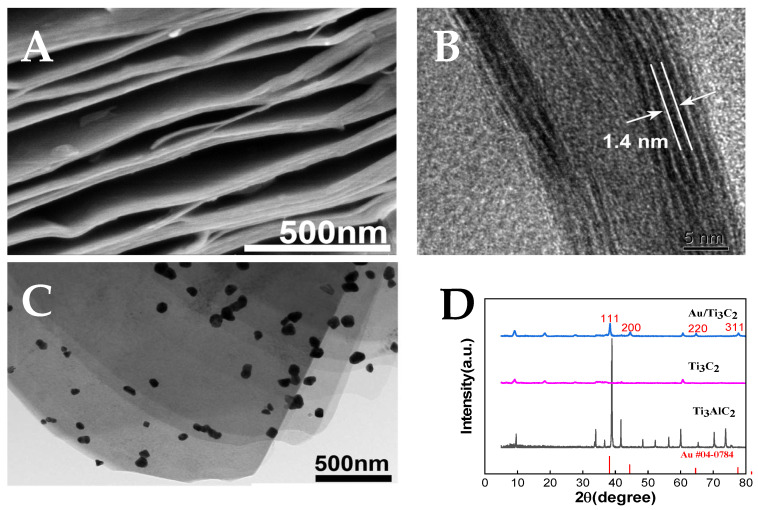
(**A**) SEM image of Ti_3_C_2_; (**B**) high-resolution transmission electron microscopy (HRTEM) image of Ti_3_C_2_; (**C**) TEM image of Au/Ti_3_C_2_; (**D**) XRD profiles of Ti_3_AlC_2_, Ti_3_C_2_, and Au/Ti_3_C_2_.

The morphology of MXene Ti_3_C_2_ was characterized using SEM and TEM. By etching Ti_3_AlC_2_ with HF, the multi-layer nanosheet structure shown in Figure 2A could be used to confirm the successful synthesis of MXene. As shown in Figure 2B, HR-TEM of Ti_3_C_2_ shows the (002) crystal face corresponding to MXene with a lattice spacing of 1.4 nm [49]. The TEM image of Au/Ti_3_C_2_ (Figure 2C) shows the multi-layer Ti_3_C_2_ decorated with AuNPs. In addition, the XRD patterns (Figure 2D) of Ti_3_AlC_2_, Ti_3_C_2_, and Au/Ti_3_C_2_ from 5° to 80° clearly show that the strongest diffraction peak at 38.4° (corresponding to the 104 plane of Ti_3_C_2_) disappeared, indicating that the Al layer in Ti_3_AlC_2_ was removed after HF etching, and Ti_3_C_2_ was obtained. For Au/Ti_3_C_2_, the XRD patterns showed peaks corresponding to the (111), (200), (220), and (311) planes of face-centered cubic Au single crystals at 38.1°, 44.3°, 64.5°, and 77.5°, respectively, and Au/Ti_3_C_2_ nanocomposites were obtained [50].

**Figure 3:**
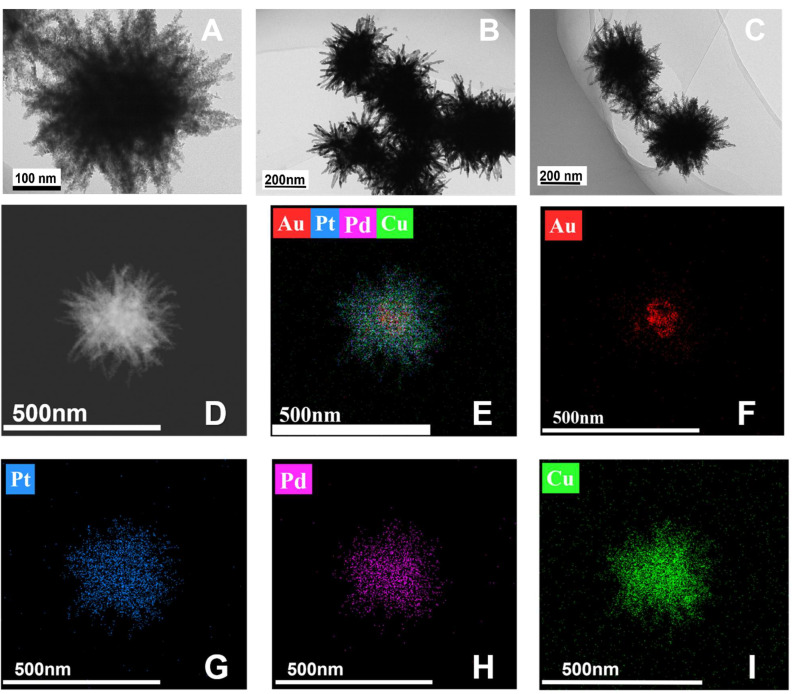
AuPtPdCu TEM images at different magnifications (**A**–**C**), and elemental maps of AuPtPdCu (**D**–**I**).

Figure 3A–C presents TEM images of the AuPtPdCu NPs. It can be seen that the prepared AuPtPdCu NP is in the shape of a three-dimensional sea urchin. According to the element mapping (Figure 3D–I), Au was mainly distributed on the inner part of the sea urchin-like structures, while Pt (blue signal), Pd (purple signal), and Cu (green signal) were dispersed throughout the structure, as can be seen from the three-dimensional structure of the AuPtPdCu NPs. Most of the Pd and Pt atoms can be observed on the branches of the nanoparticles. This multi-metallic configuration is expected to significantly enhance the catalytic activity of the nanoparticles.

According to the analysis, urchin-like AuPtPdCu may be formed through the main mechanisms of nucleation, anisotropic growth, and Ostwald maturation [51]. Firstly, the precursors (AuCl^4−^, PtCl_6_^2−^, PdCl_4_^2−^, and Cu^2+^) were respectively reduced to Au, Pt, Pd, and Cu atoms with AA. Then, when the concentrations of Au, Pt, Pd, and Cu atoms reach a certain level, they join together to form AuPtPdCu nuclei. Poly(ethylene oxide) acts as a structure-guiding agent to form AuPtPdCu NPs at certain concentrations [52].

### 3.2. Electrochemical Properties of the Immunosensor

**Figure 4:**
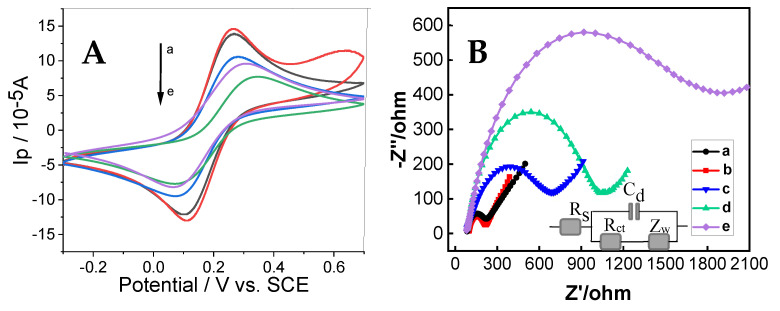
(**A**) CVs and (**B**) Nyquist plots of GCE (a), Au/Ti_3_C_2_/GCE (b), Apt/Au/Ti_3_C_2_/GCE (c), exosome/Apt/Au/Ti_3_C_2_/GCE (d), and AuPtPdCu-Apt/exosome/Apt/Au/Ti_3_C_2_/GCE (e) in 0.10 M KCl containing 5.0 × 10^−3^ M K_3_[Fe(CN)_6_]/K_4_[Fe(CN)_6_]. Inset shows the equivalent circuit.

CV and EIS are effective tools for examining the interfacial structures of immunosensors. Three electrochemical parameters—namely, peak-to-peak separation (ΔE), heterogeneous electron transfer constant (k^0^), and charge transfer resistance (R_ct_)—are presented in Table 1 [53]. As shown in Figure 4A, an increase in the redox peak current was observed after Au/Ti_3_C_2_ modification of GCE (curve b), compared to that of bare GCE (curve a), indicating that Au/Ti_3_C_2_/GCE has better electrical conductivity, thus accelerating the electron transfer between Fe(CN)_6_^3−/4−^ and the interface, as demonstrated by the calculated K^0^. This was observed as the 3D multi-layer Au/Ti_3_C_2_ modified on the GCE can improve the electroactive area of the immunosensor, leading to a higher current. When the immunosensor was fabricated with the aptamer (curve c), exosomes (curve d), and AuPtPdCu-Apt (curve e), the peak current decreased gradually with decreasing peak-to-peak separation, which was due to the non-conductivity of the aptamer and exosome molecules. The fabrication process of these insulative molecules hindered the transfer of electrons between Fe(CN)_6_^3−/4−^ and the active site of the immunosensor, which resulted in a decrease in the redox peak currents.

Figure 4B shows the EIS results of the immunosensor at each preparation step. The impedance spectra are composed of a head (semicircle) and a tail (linear), which correspond to the electron transfer and diffusion processes, respectively. The semicircle’s diameter is equal to the electron transfer resistance R_ct_ [27]. Au/Ti_3_C_2_/GCE (curve b) had a smaller R_et_ when compared with bare GCE (curve a), which can be attributed to the better conductivity of the Au/Ti_3_C_2_/GCE. Subsequently, when the aptamer (curve c), exosome (curve d), and AuPtPdCu-Apt (curve e) were gradually fabricated on the immunosensor, the R_ct_ value increased. This is due to the shielding effect of the assembled molecules. The poorer the conductivity of the molecules assembled, the great the electron transfer that will be blocked on the redox probe. These experimental results demonstrate that the immunosensor had been effectively fabricated, consistent with previous studies [54,55]. The electrochemical performance of GCE modified with AuPtPdCu NPs, AuPtPd NPs, and AuPt NCs was evaluated (Figure S1). Under identical conditions, the redox peaks of the GCE modified with AuPtPdCu NPs were significantly higher than that of those modified with AuPtPd NPs and AuPt NCs. These findings indicate that the AuPtPdCu NPs provide a greater number of active sites, thereby facilitating electron transfer and ultimately enhancing catalytic activity.

### 3.3. Optimization of the Detection Conditions

**Figure 5:**
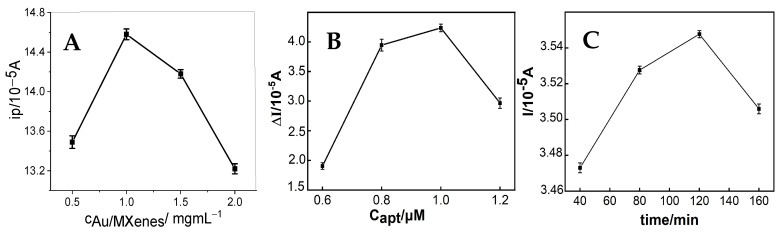
Effect of the concentration of Au/Ti_3_C_2_ (**A**), concentration of the aptamer (**B**), and incubation time (**C**) on the DPV response during the detection of exosomes.

Various parameters, including the Au/Ti_3_C_2_ concentration, aptamer concentration, and incubation time, significantly influence the experimental outcomes. Consequently, these conditions were systematically optimized to enhance the reliability and accuracy of the detection conditions of the immunosensor proposed in this study. From Figure 5A, it can be seen that the Au/Ti_3_C_2_ concentration is an important factor that influences the behavior of the immunosensor. When the concentration of Au/Ti_3_C_2_ increased from 0 to 1.0 mg mL^−1^, the current response of the immunosensor increased during exosome detection, indicating that more exosomes were captured by the aptamer loaded onto Au/Ti_3_C_2_. However, the peak current decreased when the concentration of Au/Ti_3_C_2_ exceeded 1.0 mg mL^−1^ due to the increased thickness of the Au/Ti_3_C_2_ film, which hindered electron transfer. Therefore, 1.0 mg mL^−1^ Au/Ti_3_C_2_ was selected for the following experiments.

The concentration of the aptamer also affected the signal of the immunosensor. As shown in Figure 5B, the voltammetric current of the immunosensor increased when the aptamer concentration increased from 0.6 μM to 1.0 μM, and appeared to decrease when the aptamer concentration continued to increase. This indicates that at an aptamer concentration of 1.0 μM, the aptamer achieves surface saturation on the immune sensor, thereby maximizing its exosome binding capacity. Therefore, the optimal aptamer concentration was chosen as 1.0 μM in this study.

In addition, the incubation time also influenced the immunosensor signal (Figure 5C). As shown in Figure 5C, 120 min was the optimal incubation time, with which the immunosensor obtained the highest exosome detection signal. This indicated that the binding between the exosomes and aptamers was saturated at 120 min. Therefore, the optimal incubation time for this immunoassay was 120 min.

### 3.4. Quantitative Determination of Exosomes

**Figure 6:**
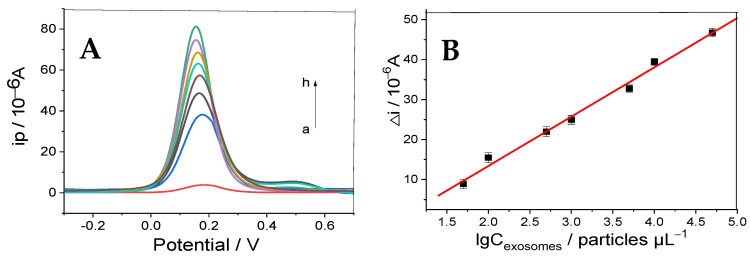
(**A**) DPV curves of the proposed immunosensor after incubation with various concentrations of exosomes in [Fe(CN)_6_]^4−/3−^ (from a to h, the exosomes concentrations are: 0, 5.0 × 10^1^, 1.0 × 10^2^, 5.0 × 10^2^, 1.0 × 10^3^, 5.0 × 10^3^, 1.0 × 10^4^, and 5.0 × 10^4^ particles µL^−1^); (**B**) linearity between the corresponding ∆I of the immunosensor and the logarithm of the exosome concentration (n = 3).

The designed electrochemical immunosensor was used to detect exosomes, in particular, using the electrochemical signal of the Fe(CN)_6_^3−/4−^ electroactive probe under optimal experimental conditions. Figure 6A shows the intensity of the DPV curve of the biosensor at different exosome concentrations. The curves from a to h in Figure 6A represent the current responses of the prepared immunosensor after incubation with exosomes of different concentrations (i.e., 0, 5.0 × 10^1^, 1.0 × 10^2^, 5.0 × 10^2^, 1.0 × 10^3^, 5.0 × 10^3^, 1.0 × 10^4^, and 5.0 × 10^4^ particles μL^−1^, respectively). The current increased with increasing exosome concentration. This result can be interpreted as follows: in the absence of exosomes, aptamers can conjugate and interact with the Au/Ti_3_C_2_ fixed on the working electrode, thereby diminishing the electrochemical response of [Fe(CN)_6_]^3−/4−^. Indeed, the formation of immune complexes in solution does not alter the active surface area of the electrode due to the presence of exosomes (by the time these complexes reach the working electrode surface, the aptamer binding sites are already occupied). Consequently, a higher concentration of exosomes should result in a greater current response. Taking the exosome concentration in Figure 6A as a logarithm, the relationship between it and the peak current is shown in Figure 6B, which clearly shows that the logarithmic value of the exosome concentration is proportional to the redox peak current in the range of 5.0 × 10^1^ to 5.0 × 10^4^ particles μL^−1^. The linear regression equation was ∆I = 1.234 × 10^−5^ logCexosomes − 1.129 × 10^−5^ (particles μL^−1^), with a correlation coefficient of 0.9949, and the limit of detection of exosomes was 19 particles μL^−1^ (S/N = 3). The analytical performance of the studied immunosensor was compared with our laboratory’s previously reported results. Both displayed a similar linear range (50–5.0 × 10^4^ particles μL^−1^, 100–5 × 10^5^ particles μL^−1^), possibly due to a limitation of the immunosensor designed with MXenes composites. Moreover, when the results of the developed sandwich immunosensor for exosome detection were compared with other data reported by previous researchers (Table 2), it can be seen that the designed signal amplification strategy outperforms similar methods. This can be attributed to the excellent construction of the immunosensor. First, Au/Ti_3_C_2_ is used as the substrate material, which has a large specific surface area with good biocompatibility, thus providing more active sites to capture the aptamer. Second, the sea urchin-like three-dimensional structure of AuPtPdCu leads to excellent catalytic properties that improve its sensitivity. The obtained results indicate that the immunosensor can quantitatively detect exosomes in clinical applications, as demonstrated through analysis of the experimentally derived results.

### 3.5. Specificity, Reproducibility, and Stability of the Immunosensors

**Figure 7:**
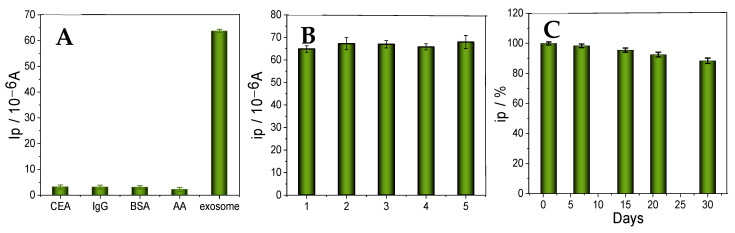
(**A**) Specificity of the immunosensor for various interferers, CEA, IgG, BSA, and AA, at a concentration of 10 ng mL^−1^. (**B**) Reproducibility of the immunosensor (five independent immunosensors, exosome concentration: 5.0 × 10^2^ particles μL^−1^, error bars represent standard deviation, n = 3). (**C**) Stability of the immunosensor.

Specificity is vital for immunosensors. The selectivity was analyzed by adding interferers, such as CEA, IgG, BSA, and AA. As shown in Figure 7A, the developed immunosensor had good selectivity, as the signal value of exosomes (5.0 × 10^2^ particles µL^−1^) was significantly higher than that of the interferers (10 ng mL^−1^). Figure 7B shows the reproducibility of the immunosensor. For this purpose, five independent immunosensors were applied to detect exosomes (5.0 × 10^2^ particles µL^−1^) under optimized conditions. The results showed that the immunosensor had good reproducibility, with a relative standard deviation of 3.17%.

To investigate the stability of the constructed sandwich-type immunosensor, it was stored at 4 °C for several days, then taken out to measure the electrochemical peak current. As shown in Figure 7C, the electrochemical measurements decreased by less than 10% within three weeks. Therefore, its stability is good; however, as stability detection was not performed when storing it at room temperature, the immunosensor using Au/MXene as the platform needs further improving.

### 3.6. Detection of Real Samples

To investigate the application of the prepared immunosensor to biological samples, colorectal-derived exosomes in different concentrations were added to diluted human serum samples (where the human serum was diluted five times with PBS) for detection using a standard addition method. The results shown in Table 3 indicate that the recovery of exosomes ranged from 94.7% to 103.5%, indicating that the developed immunosensor has an acceptable accuracy for the detection of exosomes and can be used for clinical diagnosis.

## 4. Conclusions

In summary, an electrochemical sandwich immunosensor for exosome analysis was realized using multi-layer Au/Ti_3_C_2_ nanoparticles and CD63 aptamer-conjugated AuPtPdCu composites. The multi-layer Au/Ti_3_C_2_ nanoparticles can attract more aptamers to obtain exosomes, considering their large surface area and excellent electrical conductivity. The AuPtPdCu composites improved the detection signal due to the excellent synergistic effects of the used micro-nanomaterials. Under optimized conditions, the immunosensor exhibited a wide linear range (5.0 × 10^1^ to 5.0 × 10^4^ particles µL^−1^) and a low limit of detection (LOD = 19 particles µL^−1^, S/N = 3) in the context of exosome detection. Additionally, the proposed electrochemical immunoassay exhibited excellent reproducibility, specificity, and stability. Moreover, exosome detection testing in serum samples yielded satisfactory results. Therefore, as a rapid and user-friendly point-of-care tool, this immunosensor can be clinically utilized to monitor the presence and progression of colorectal tumors through the detection of tumor-derived exosomes. Furthermore, due to its simplistic design, the immunosensor can be readily adapted for the detection of various other medically relevant diagnostic biomarkers through modification of the specific bioreceptor.

## Figures and Tables

**Table 1:** Electrochemical parameters of peak-to-peak separation (ΔE), heterogeneous electron transfer rate constant (k^0^), and charge transfer resistance (R_ct_).

Fabrication Step	CV		EIS	
	ΔE/V	K^0^/cm s^−1^	R_ct_/KΏ	K^0^/cm s^−1^
Bare	0.18 ± 0.02	—	0.15 ± 0.2	—
Au/Ti_3_C_2_	0.17 ± 0.02	2.7 (±0.1) × 10^−2^	0.11 ± 0.2	3.13 ± (0.2) × 10^−3^
Apt/Au/Ti_3_C_2_	0.20 ± 0.02	—	0.5 ± 0.4	—
Exosome/Apt/Au/Ti_3_C_2_	0.22 ± 0.02	—	1.1 ± 0.5	—
Apt/AuPtPdCu/exosome/Apt/Au/Ti_3_C_2_	0.24 ± 0.01	—	1.8 ± 0.2	—

**Table 2:** Comparisons of the detection ranges and detection limits of various exosome detection methods.

Method	Matrix	Detection Range (Particles μL ^−1^)	Detection Limit (Particles μL^−1^)	Refs.
Fluorescence	Graphene oxide–DNA aptamer	3.0 × 10^4^ to 6.0 × 10^5^	2.1 × 10^4^	[56]
Fluorescence	Biotin-functionalized phosphatidylethanolamine	4.0 × 10^3^ to 2.0 × 10^5^	2.0 × 10^3^	[57]
Electrochemiluminescence	CdS quantum dots in the inner pores of DNA microcapsules	2.0 × 10^2^ to 7.5 × 10^4^	60	[58]
Electrochemiluminescence	Lum-AuNPs@g-C_3_N_4_	10^2^ to 10^7^	39	[59]
Electrochemistry	Cucurbit [7] uril modified gold and ferrocene	5.0 × 10^2^ to 5.0 × 10^3^	4.82 × 10^2^	[55]
SERs	Gold–silver–silver core–shell–shell nanotrepangs	1 to 1.0 × 10^7^	35	[60]
Fluorescence	Black phosphorus (BP)@Mn^2+^/DNA	1.0 × 10^5^ to 1.0 × 10⁶	2.5 × 10^4^	[61]
Electrochemiluminescence	Zirconium-based conjugated polymers and polyethyleneimine	10^2^ to 10^8^	33	[62]
Hydrogel microneedles extraction	Hydrogel microneedles	10^2^ to 10^6^	100	[63]
Electrochemistry	Au/MXenes and AuPtPdCu	5.0 × 10^1^ to 5.0 × 10^4^	19	this work

**Table 3:** Detection results obtained with the immunosensor.

Sample Number	Added (LgC particles μL^−1^)	Found (LgC particles μL^−1^)	Recovery (%)
1	1.69	1.698	100.4
2	2	1.895	94.7
3	2.69	2.624	97.5
4	3	3.047	101.5
5	3.39	3.510	103.5

## Data Availability

All data supporting the findings of this study are available within the article and its Supplementary Materials.

## Supplementary Materials

The following supporting information can be downloaded at: https://www.mdpi.com/article/10.3390/mi16030280/s1, Figure S1: CV curves of GCE modified with AuPtPdCu NPs, AuPtPd NPs and AuPt NCs.
