# Supplementary figures and images for: An Electrochemical Immunosensor for Sensitive Detection of Exosomes Based on Au/MXenes and AuPtPdCu
Source: Micromachines (Basel). 2025 Feb 27;16(3):280. doi: 10.3390/mi16030280 (PMC11944654; doi:10.3390/mi16030280)

## Supplementary File

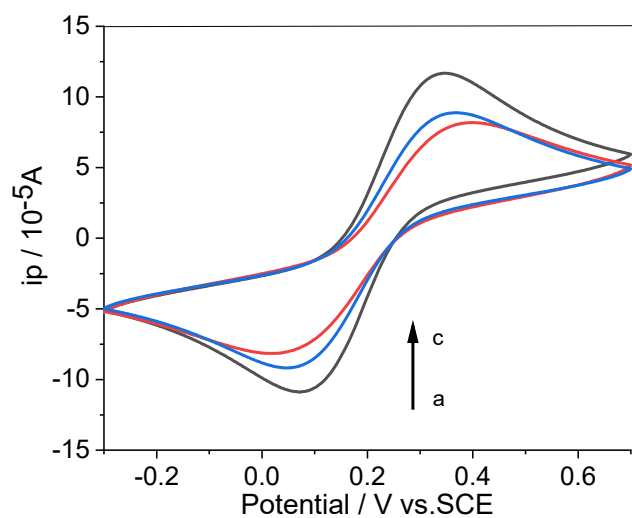

Figure S1 CV curves of GCE modified with AuPtPdCu NPs, AuPtPd NPs and AuPt NCs.

Supplement: Supplementary file 1 [file micromachines-16-00280-s001.zip › micromachines-3481089-supplementary.pdf]
